# Narasin used as a feed additive in conventional rearing of broilers can co-select for vancomycin-resistant *Enterococcus faecium* through the NarAB ionophore resistance mechanisms

**DOI:** 10.1093/jac/dkag253

**Published:** 2026-08-25

**Authors:** Rikki Franklin Frederiksen, Silje Granstad, Ida Thøfner, Jannice Schau Slettemeås, Karin Lagesen, Anne Margrete Urdahl, Roger Simm

**Affiliations:** Department of Animal Health and Food Safety, Norwegian Veterinary Institute, Ås, Norway; Department of Biosciences, University of Oslo, Oslo, Norway; Department of Animal Health and Food Safety, Norwegian Veterinary Institute, Ås, Norway; Department of Veterinary and Animal Sciences, University of Copenhagen, Copenhagen, Denmark; Department of Animal Health and Food Safety, Norwegian Veterinary Institute, Ås, Norway; Department of Animal Health and Food Safety, Norwegian Veterinary Institute, Ås, Norway; Department of Animal Health and Food Safety, Norwegian Veterinary Institute, Ås, Norway; Department of Biosciences, University of Oslo, Oslo, Norway

## Abstract

**Objectives:**

To investigate the role of the NarAB resistance mechanism in the selection of vancomycin-resistant *Enterococcus faecium* (VREfm) and assess the impact of ionophore feed additives, particularly narasin, on the emergence of VREfm in broiler chickens.

**Materials and methods:**

Three isogenic *E. faecium* strains with different antimicrobial resistance determinants were created by mutagenesis and conjugation and used in a controlled animal experiment. Ross 308 broiler chickens were inoculated with either a rifampicin-resistant, a rifampicin- and vancomycin-resistant or a rifampicin-, vancomycin- and narasin-resistant strain and fed diets supplemented with selected ionophores. Bacterial populations were analysed on selective Slanetz and Bartley agar to determine the presence and selection of VREfm and other vancomycin-resistant species. Bacterial inoculation strains and isolates were whole genome sequenced for species identification and to identify genetic resistance mechanisms.

**Results:**

Narasin was shown to select for VREfm in broilers, with NarAB being essential for co-selection. Intrinsically vancomycin-resistant *Pediococcus acidilactici* and *Enterococcus gallinarum* were identified as part of the broilers’ vancomycin-resistant resident microbiota. Notably, among the *P. acidilactici* isolates that were susceptibility tested, strains resistant to both vancomycin and narasin were only found in broilers fed narasin, supporting that narasin promotes the growth of narasin-resistant populations.

**Conclusion:**

Narasin use in broiler feed can co-select for vancomycin-resistant bacteria, including VREfm, through the NarAB mechanism. These findings emphasize the concerns associated with the use of particular ionophores in poultry and suggest that vancomycin and narasin resistance may be more widespread in the broiler microbiota than previously recognized. Further research is needed to understand the implications for antimicrobial resistance and human health.

## Introduction

*Enterococcus faecium* is an important opportunistic pathogen that can cause serious infections in humans. These bacteria often display resistance to multiple antimicrobials, which complicates treatment options and increases healthcare costs.^1^ Vancomycin-resistant *E. faecium* (VREfm) is listed by the WHO as a High Priority Pathogen requiring further research for effective prevention and control.^2^ VREfm infections continue to increase in many countries despite antimicrobial stewardship in human healthcare,^3^ highlighting the need for broader measures including control in animals and the environment.^4^

VRE was repeatedly reported in animal populations in the 1990s.^5–7^ This was linked to cross-resistance between vancomycin and avoparcin, a growth promoting glycopeptide used in feed.^5,8^ Following a ban on avoparcin in Norway and Denmark in 1995, and the EU in 1997, occurrence of VRE in broilers and pigs gradually decreased,^9–11^ but selective methods for detection show that VRE has persisted in poultry populations 20–25 years after the ban.^12–14^

Although the reason for VRE persistence in animal populations remains unclear,^15,16^ several contributing factors have been suggested, including incomplete eradication between flocks, plasmid stability, frequent horizontal gene transfer and host adaptation.^12,17,18^ Ionophore feed additives, which are used to control coccidiosis, have also been considered as contributing factors.^17,19,20^ The use of ionophore feed additives overlapped with the use of avoparcin and today ionophores are the main antimicrobial feed additives used in poultry. An ionophore resistance mechanism encoded by the *narAB* genes was first discovered in VREfm from Swedish broilers.^21^ NarAB is an ABC-type membrane transporter that confers resistance to the ionophores salinomycin, narasin and maduramicin, but not to monensin and lasalocid.^22^ The *narAB* genes have been found in VREfm from poultry in Norway, Denmark and Sweden^23^ and occur on pVEF-like mobilizable plasmids, which also carry the *vanA* gene cluster conferring vancomycin resistance on Tn*1546* type transposons.^5,12,17,21,24^ Co-localization of vancomycin resistance and ionophore resistance suggests co-selection of resistance. Narasin was discontinued as a prophylactic feed additive in Norway in 2015–2016 and VRE has since then been below the detection limit of the selective method used in the Norwegian surveillance programme for food, feed and animals.^25,26^ By contrast, Sweden continues to use narasin and VREfm were detected in 5% of samples in 2020 using the same selective method as used in Norway.^13^ These findings indicate that narasin supplementation of broiler feed and co-localization of narasin and vancomycin resistance contribute to VREfm selection. However, the narasin-mediated co-selection of vancomycin resistance and potential human health implications remains under discussion.^15,16,27,28^ This study provides the first *in vivo* evidence from a controlled animal experiment that narasin selects for VREfm and that NarAB is required for co-selection.

## Materials and methods

### Bacterial strains, media and antimicrobials

The bacterial strains used in this study are listed in Table 1. Bacteria were grown on blood agar, Slanetz and Bartley (SB) agar, Luria-Bertani (LB) agar or in Luria-Bertani medium. Media were supplemented with vancomycin (4 mg/L), narasin (0.5 mg/L) or rifampicin (16 mg/L) when required for selection of antimicrobial resistance.

**Table 1.** Bacterial strains and plasmids used in this study

Strain	Resistance	Reference
*E. faecium* SVARM 2000-172	Van^R^, Nar^R^	^29^
*E. faecium* SVARM 2001-233	Van^R^, Nar^R^	^29^
*E. faecium* WT1428		This study
*E. faecium* WT1428-R	Rif^R^	This study
*E. faecium* TC172	Rif^R^, Van^R^	This study
*E. faecium* TC233	Rif^R^, Van^R^, Nar^R^	This study

Van^R^, vancomycin resistant; Nar^R^, narasin resistant; Rif^R^, rifampicin resistant

### Construction of inoculation strains

*E. faecium* (WT1428) isolated from Norwegian poultry in 2002 was used to generate the inoculation strains. A rifampicin-resistant mutant (WT1428-R) was produced via incremental exposure to rifampicin (0.03 to 16 mg/L) and selection on LB agar with rifampicin (64 mg/L). Sequencing confirmed the presence of mutations conferring rifampicin resistance.

A rifampicin- and vancomycin-resistant *E. faecium* strain (TC172) and a strain also resistant to narasin (TC233) were generated via filter mating as previously described^22^ using WT1428-R as recipient and SVARM 2000-172 and SVARM 2001-233 as donors. In short, donors and recipient were grown in LB with appropriate antimicrobials, mixed (9:1), spotted on LB agar and incubated overnight. Transconjugants were selected on LB agar with rifampicin and vancomycin, then patched onto plates with narasin and vancomycin or vancomycin alone. The constructed inoculation strains were sequenced to confirm transfer of resistance determinants.

### Antimicrobial susceptibility testing

Antimicrobial susceptibility of the donor, recipient and transconjugant strains was analysed by broth microdilution. Colonies grown on blood agar (37°C, 18 h) were suspended to 0.5 McFarland in cation-adjusted Mueller Hinton Broth (CAMBHT) and 50 µL was added to 96-well plates. The Sensititre^TM^ EU surveillance Enterococcus EUVENC AST panel (Thermo Fisher Scientific Inc, Waltham, MA, USA) tested susceptibility to 12 antimicrobials, including vancomycin. Ionophore susceptibility (narasin, lasalocid (Sigma-Aldrich, St. Louis, MO, USA); 0.03–32 mg/L) was tested separately using broth microdilution in 96-well microtitre plates. Narasin and lasalocid were chosen as examples of ionophores that do and do not select for *narAB* positive *E. faecium in vitro*, respectively. Plates were incubated (35°C, 18 h) and read using a BIOMIC V3 system (Giles Scientific Inc., Santa Barbara, CA, USA). Strains were classified as resistant if the MIC exceeded the EUCAST epidemiological cut-off (ECOFF) values: vancomycin >4 mg/L, narasin >0.5 mg/L and lasalocid >2 mg/L.^30^

### Sequencing of bacteria

DNA was extracted using QIAamp DNA Mini Kit (Qiagen, Germany) according to the manufacturer’s instructions for bacterial DNA extraction with a minor modification in that lysostaphin was replaced with 2500 U/mL mutanolysin from *Streptomyces globisporus* ATCC 21553 (Sigma-Aldrich, USA). The concentration and quality of the DNA was measured by the Qubit dsDNA BR assay (Thermo Fisher Scientific Inc, Waltham, MA, USA) and Nanodrop (Thermo Fisher Scientific Inc.), respectively. DNA with concentration above 20 µg/mL, and A260/A280 and A260/A230 ratios above 1.8 and 1.4, respectively, was sequenced. The DNA was stored at −20°C.

For long-read sequencing of bacteria DNA was barcoded with the SQK-RBK114.24 ligation sequencing kit and sequenced using a MinION Flow Cell R10.4.1 on a MinION sequencer (Oxford Nanopore Technologies, UK). Base calling was done with Guppy version 6.5.7^31^ with a minimum quality score of 7 and using the model dna_r10.4.1_e8.2_260bps_sup.cfg. For short-read sequencing of bacteria, sequencing libraries from DNA extracts were prepared using the Illumina DNA Prep kit (Illumina, USA) and sequencing was performed on an Illumina MiSeq instrument, producing paired-end reads of 300 bp.

### Bioinformatic analysis

Short reads were quality controlled and *de novo* assembled using the Bifrost pipeline^32^ which includes quality control (fastQC version 0.11.9^33^ and MultiQC version 1.9^34^), stripping PhiX (BBDuk version 38.76^35^), trimming (Trimmomatic version 0.39^36^), assembly (SPAdes version 3.14.0^37^), polishing (Pilon version 1.23^38^) using untrimmed reads mapped to the assembly with BWA version 0.7.8^39^ and the assembly quality was assessed using QUAST version 5.2.0.^40^

Long reads were demultiplexed with Qcat version 1.1.0^41^ with a minimum read length of 50, filtered with Nanofilt version 2.8.0^42^ using a minimum quality score of 9 and a minimum read length of 300. Finally, filtered reads were assembled with Flye version 2.9^43^ with an assembly coverage of 200x.

Draft assemblies from donors, recipients and transconjugants were annotated using Bakta version 1.8.1 with the ‘–complete’ option.^44^ Chromosome and plasmid contigs were assigned MOB-recon (MOB-suite version 3.0.3^45^) and antimicrobial resistance (AMR) genes identified with ResFinder version 4.1.0^46^ and the Comprehensive Antibiotic Resistance Database (CARD)^47^ accessed on 13 January 2025 using strict, high-quality protein criteria. Circular comparative BLAST alignment of plasmids was performed with BLAST Ring Image Generator (BRIG).^48^

A pan-genome-wide association study (pan-GWAS) was performed to assess the relationship between gene presence and narasin resistance. Pangenomes were constructed using the ‘Core gene’ module of the ALPPACA pipeline version 2.0.3^49^ with annotations from Prokka version 1.14.5^50^ and analysis by Panaroo version 1.2.9.^51^ Fisher’s exact test was applied in R to gene presence/absence and narasin resistance data. Genes with a *P* < 0.05 were considered significantly associated with narasin resistance.

### Animal experiment

Three pelleted experimental feeds were produced at the Centre for Feed Technology, (NMBU, Ås, Norway); two containing narasin or lasalocid at theoretical commercial concentrations, and one control feed without coccidiostats. All met the nutritional requirements of Ross 308 broilers with consistent formulation (Table S1, available as Supplementary data at *JAC* Online). Ionophore concentrations were analysed by Eurofins Food and Feed Testing (Moss, Norway) using LC-MS/MS.

Ross 308 was chosen because intensive broiler production, characterized by very large holdings, indoor rearing, high stocking densities and fast growth rates, comprise ∼90% of the broiler production in EU and Ross 308 is one of the dominating fast growing broiler hybrids worldwide.^52^

Pre-incubated embryonated Ross 308 eggs from a commercial hatchery (Danhatch A/S, Ragebøl, Denmark) were hatched at the Department of Veterinary and Animal Sciences, University of Copenhagen, Frederiksberg C, Denmark.

A total of 105 as-hatched broilers were individually allocated to seven groups of 15 birds each and housed in separate rooms at a Biosafety Level 2 experimental animal facility at the Department of Experimental Animal Medicine, University of Copenhagen, Frederiksberg C, Denmark and were cared for by trained personnel throughout the study to ensure proper husbandry and welfare. The as-hatched chicks were allocated directly from the hatcher, blindfold, by allocating the individual chick into one of seven separate clean boxes until group size reached 15 birds. Each of the seven boxes were randomly assigned a group ID. After assigning group ID all chicks had an individual cloacal swab taken. During this swabbing, three birds from each group were chosen blindfold as focal individuals and tagged with legband with a unique ID.

**Figure 1.**
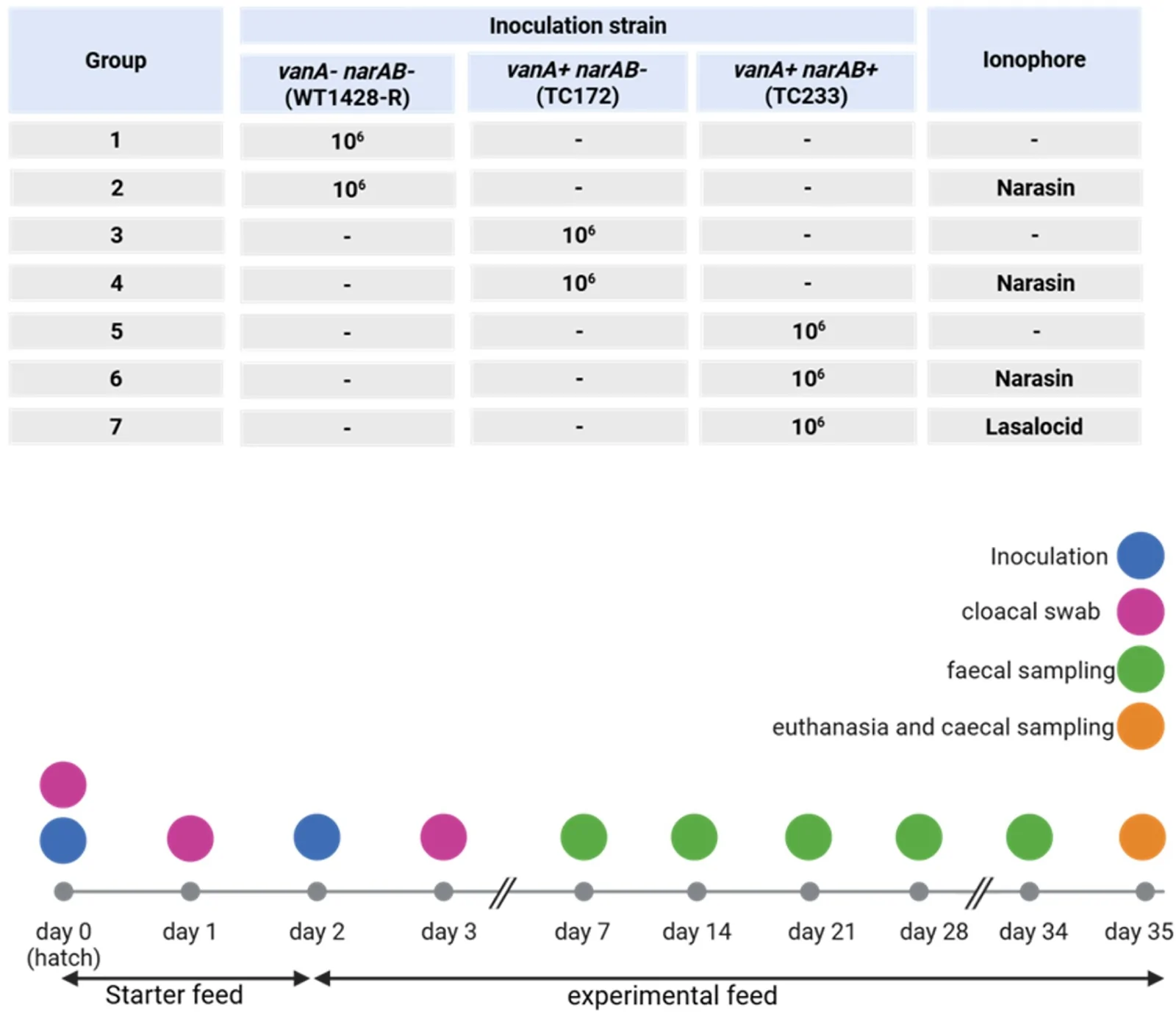
Design and timeline of animal experiment. Overview of experimental groups with their given experimental feed (Feed 1 without ionophores, Feed 2 supplemented with narasin and Feed 3 supplemented with lasalocid) and *E. faecium* inoculation strains: *vanA− narAB−*, inoculation control strain (WT1428-R); *vanA+ narAB−*, inoculation strain carrying vancomycin resistance (TC172) and *vanA+ narAB+*, inoculation strain carrying vancomycin and narasin resistance (TC233). Intended inoculum size in cfu is given in the table.

The chickens were housed in groups of 15 in pens (1.52 × 2.18 m) on clean wood shavings with stocking densities meeting the EU legislation criteria for domestic chicken on the protection of animals used for scientific purposes.^53^ The litter was left in the pens throughout the experimental period and top-dressed with additional clean wood shavings when needed for keeping litter dry. Pens included in addition to bedding, perches, dust bath and feed and water *ad libitum*. Chickens received a starter diet (Table S1) and were switched on day 3 to customized feed without ionophores (feed 1) or with narasin (feed 2) or lasalocid (feed 3) (Figure 1, Table S2). Environmental conditions followed breed and age specific guidelines.^54^

Chickens were inoculated by oral gavage with 10^6^ cfu of WT1428-R, TC172 or TC233 in a 100 µL suspension on days 0 and 2. Inoculum sizes were confirmed by plating 10-fold serial dilutions of the bacterial suspension on LB agar. Colonies were counted after incubation overnight at 37°C. To prepare the bacterial suspensions, bacteria were grown overnight at 37°C in LB with or without vancomycin, spun down, re-suspended in LB and grown to an optical density (OD) 0.1 nm and diluted in saline (0.9% NaCl) to OD 0.005 (5 × 10^6^ cfu/mL).

Cloacal swabs were taken using a small sterile cotton steel swab that were gently inserted into the cloaca and rotated to ensure good contact to the cloacal mucosa. The content of the swabs was suspended in 1 mL of buffered peptone water and the samples were 10-fold diluted in normal saline and plated on SB agar with no antimicrobials, vancomycin or vancomycin and rifampicin. Cfus were counted after incubation at 37°C for 48 h. The cloacal samples were taken on day 0 and plated on SB agar to monitor whether the chickens were free from *E. faecium* at the day of hatch. Post-inoculation, cloacal swabs taken on days 1 and 3 were streaked on SB agar with vancomycin and rifampicin to confirm colonization and detect contamination. On day 0 the cloacal swabs were taken just after assignment to groups (see previously). On day 1 and 3 the swabs were taken in the pens. At all time points, all birds were sampled. The detection limit was 200 cfu/swab.

Faecal samples were collected from the focal chickens on days 7, 14, 21, 28 and 34 post-infection by temporarily placing the focal birds individually into a clean box until faeces passed (Figure 1). The faecal sample were then weighed, 10-fold serially diluted in normal saline and plated on SB agar without antimicrobials, with vancomycin and with vancomycin and rifampicin. Cfus were counted after incubation at 37°C for 48 h. The detection limit of the method was 200 cfu/g of faeces. On day 35, 12 chickens per group were euthanized by cervical dislocation following mechanical stunning by a blow to the head. All chickens were weighed immediately after euthanasia followed by a full necropsy^55^ and the caecal content was collected. Macroscopic pathological changes were recorded if present. All remaining birds were euthanized on day 35.

Caecal content was weighed, 10-fold serially diluted in normal saline and plated on SB agar with no antimicrobials, vancomycin or vancomycin and rifampicin. Cfus were counted after incubation at 37°C for 48 h. Cfu counts on SB agar were considered a total count of bacteria. The number of resident vancomycin-resistant bacteria was estimated by subtracting cfus on vancomycin and rifampicin agar from vancomycin-only agar. Colonies on vancomycin and rifampicin agar represented TC172 or TC233 strains. The detection limit of the method was 200 cfu/g of caecal content. The identity of bacteria giving rise to colonies with different morphologies was determined using MALDI-TOF MS.

### Ethical statement

This study was approved by the Danish Animal Experiments Inspectorate (Licence: 2019-15-0201-01611).

### Statistical analyses

Group means of body weights and log-transformed caecal cfu counts were compared using one-way ANOVA, with Tukey’s HSD test (R version 4.3.0^56^) and *P* < 0.05 was considered significant. *Q–Q* plots assessed normality of log-transformed data.

## Results

### Characterization of inoculation strains

Antimicrobial susceptibility of the constructed inoculation strains was assessed by broth microdilution (Table S3). WT1428-R showed resistance to rifampicin but was susceptible to narasin and vancomycin. TC172 was resistant to rifampicin and vancomycin whereas TC233 was resistant to rifampicin, vancomycin and narasin. Whole genome sequencing of WT1428-R revealed an *rpoB* S491L mutation linked to rifampicin resistance (Table S3).^57^ TC233, carried *vanA* and *narAB* resistance genes, TC172 harboured only *vanA* (Table S3). Sequencing confirmed the presence of a plasmid from donor strains SVA 2001-233 in TC233 (Table S3). In TC172, *vanA* was located on a circular contig with plasmid-like features but not matching known MOB-suite plasmids (Table S3).

### Customized feed

Ionophore concentrations in customized feeds were measured by LC-MS/MS (Table 2). Narasin in Monteban^®^-supplemented feed matched the expected level (67 ppm), while lasalocid in Avatec^®^-supplemented feed was lower than expected (17 ppm; 19% of theoretical concentration).

**Table 2.** Theoretical and measured ionophore concentrations in experimental feeds

	Theoretical concentration (ppm)			Measured concentration (ppm)		
	Feed 1	Feed 2	Feed 3	Feed 1	Feed 2	Feed 3
Narasin	0	70	0	<0.05	67	<5.0
Lasalocid	0	0	90	<0.05	<5.0	17

Values reported as < indicate concentrations below the limit of quantification of the analytical method.

### Animal experiment

**Figure 2.**
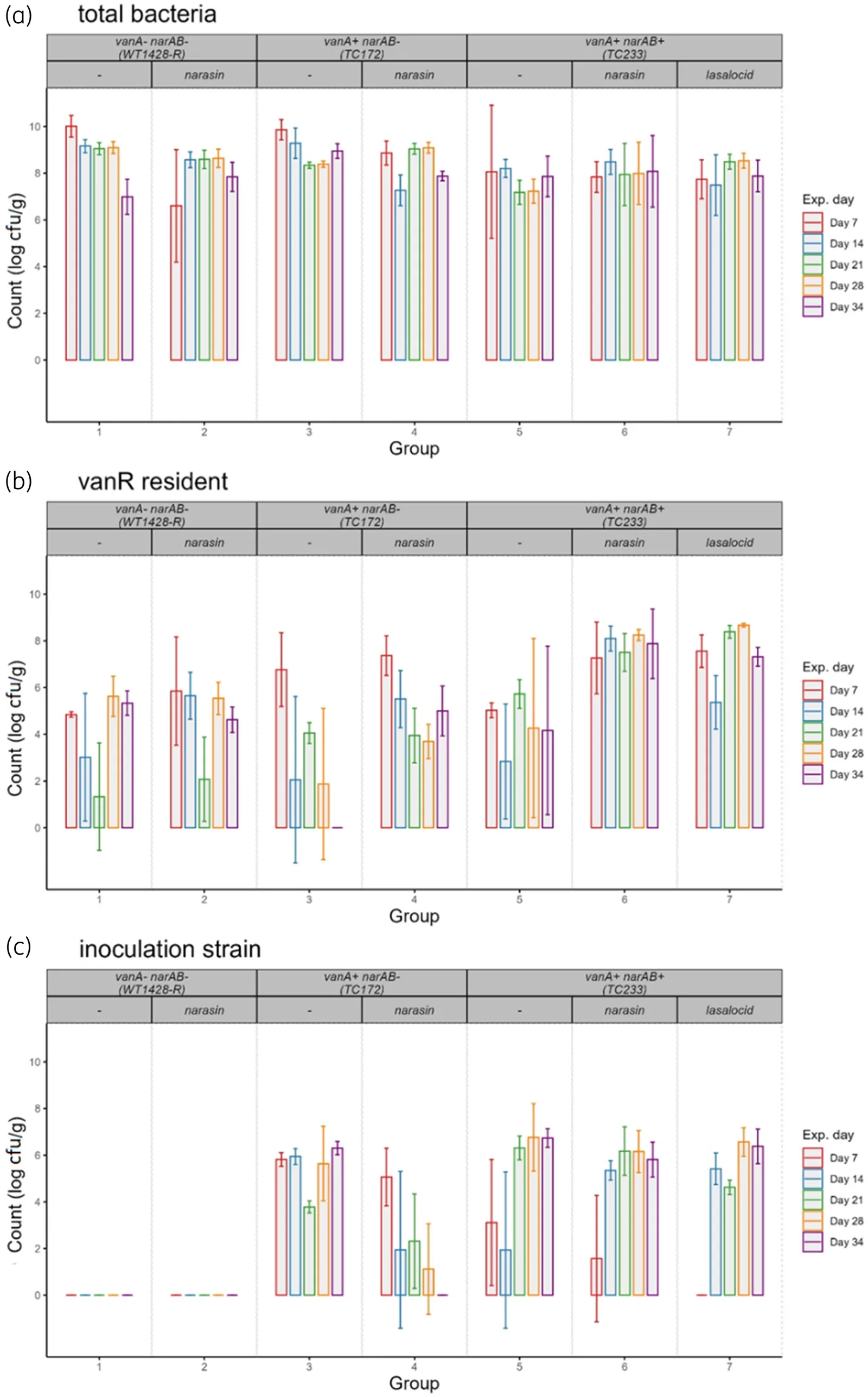
Number of cfu in faecal content from broilers fed diets with and without ionophore supplementation sampled over the course of the experiment. (A) Total bacterial count corresponds to the number of cfu on SB agar without antimicrobial supplementation. (B) VanR resident refers to the number of bacteria growing on SB agar supplemented with vancomycin with the number of bacteria on SB agar supplemented with vancomycin and rifampicin subtracted. (C) Inoculum refers to the number of cfu on SB agar with vancomycin and rifampicin. The charts are divided into groups of broilers receiving similar inoculation strain (upper legend) and feed (lower legend) as indicated by the legends above the chart. *vanA− narAB−* (WT1428-R), *vanA+ narAB−* (TC172) and *vanA+ narAB+* (TC233), (feed without ionophores), narasin (feed supplemented with narasin) and lasalocid (feed supplemented with lasalocid). Bars are means of log-transformed counts of cfu from three focal chickens in each group, and error bars are standard deviations.

On day 0 cloacal swabs showed no cfu on SB agar, indicating enterococcal colonization was below detection limit. Swabs from days 1 and 3 confirmed presence of inoculation strains in groups 3–7, while groups 1 and 2 remained negative on SB agar with vancomycin and rifampicin. Faecal samples were regularly cultured on SB agar variants (Figure 1). Total bacterial counts remained stable across groups (Figure 2A). Vancomycin-resistant, rifampicin-sensitive resident bacteria were found in all groups, with variable levels over time (Figure 2B). Group 3 showed a decline from day 7 to 34, group 4 remained stable, other groups showed little change. As expected, no vancomycin- and rifampicin-resistant bacteria were found in groups 1 and 2 (Figure 2C). In group 3, inoculation strain levels were stable, group 4 showed a decline to undetectable levels by day 34, groups 5–7 showed increasing levels over time.

**Figure 3.**
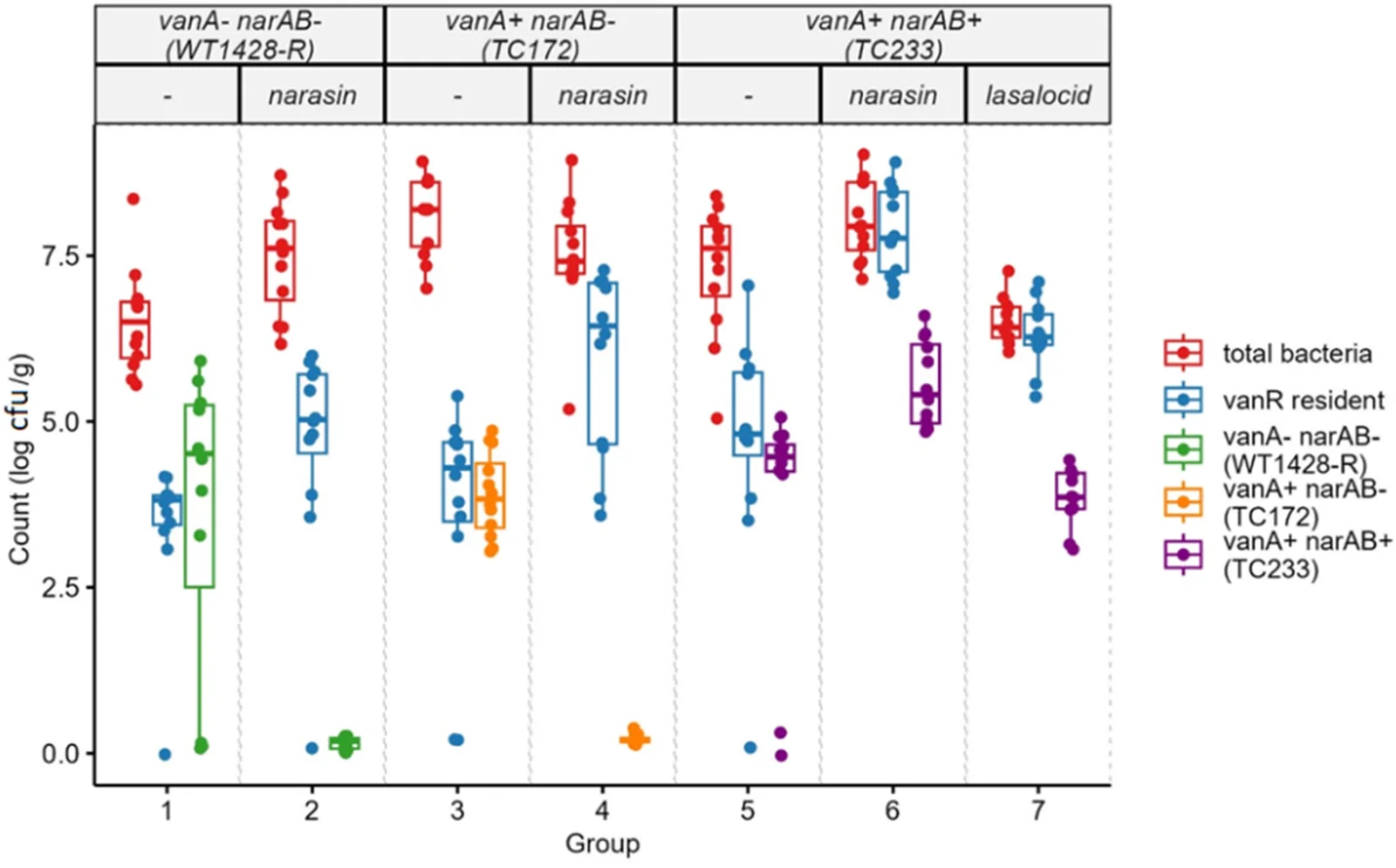
Number of cfu in caecal content from broilers fed diets with and without ionophore supplementation on day 35. The charts are divided into groups of broilers receiving similar inoculation strain (upper legend) and feed (lower legend) as indicated by the legends above the chart*. vanA− narAB−* (WT1428-R), *vanA+ narAB−* (TC172) and *vanA+ narAB+* (TC233), (feed without ionophores), narasin (feed supplemented with narasin) and lasalocid (feed supplemented with lasalocid). Total bacterial count corresponds to the number of cfu on SB agar without antimicrobial supplementation. The *vanA− narAB−* strain was sampled on SB agar supplemented with rifampicin, whereas the *vanA+ narAB−* and *vanA+* and *narAB+* strains were counted on SB agar supplemented with vancomycin and rifampicin. VanR resident refers to the number of bacteria growing on SB agar supplemented with vancomycin with the number of bacteria on SB agar supplemented with vancomycin and rifampicin subtracted. The middle line of the boxes represents the median of log-transformed cfu counts per gram caecal content, while upper and lower edges of the box represent first quartile and third quartile, respectively. Whiskers extend to data points within 1.5 times the IQR from the edges of the box.

Broiler weights on day 35 ranged from 1911 to 3291 g with no significant group differences (Table S4). Post-euthanasia, caecal cfu counts were assessed (Figure 3). Groups 1 and 7 had significantly lower cfu on unsupplemented SB agar than groups 2–6 (Table 3). For strain TC172 (rifampicin and vancomycin resistant and narasin susceptible), cfu counts were higher in group 3 than group 4. TC233 (resistant to vancomycin, rifampicin and narasin) showed significantly more cfu in group 6 than in groups 5 and 7, with no difference between the last two. WT1428-R (rifampicin resistant, vancomycin and narasin susceptible) showed no cfu on SB agar with rifampicin and vancomycin, and fewer cfu on rifampicin-only agar in group 2 compared to group 1. Most groups had higher cfu on vancomycin-only agar than on rifampicin and vancomycin agar, except groups 3 and 5 that had similar levels on both types of agar. Resident vancomycin-resistant cfu were higher in group 4 than group 3 and in groups 6 and 7 compared to group 5 (Figure 3, Table 3).

**Table 3.** Description of statistical differences in bacteria from caecal contents of broilers

	Group						
	1	2	3	4	5	6	7
Total bacteria	b	a	a	a	ab	a	b
VanR resident	bc	bc	c	ab	bc	a	a
*vanA−, narAB−*	a	b					
*vanA+, narAB−*			a	b			
*vanA+, narAB+*					b	a	b

Letters in each row indicate statistical significance (*P* < 0.05) between groups as determined using one-way ANOVA and Tukey’s HSD test and groups sharing the same letter indicate that there is no difference between these groups (*P* > 0.05). The underlying data are shown in Table S5.

VanR resident, vancomycin-resistant resident bacteria; *vanA*, vancomycin-resistance operon; *narAB*, narasin resistance operon; *−*, absence of; +, presence of.

Owing to animal welfare reasons, two chickens from group 6 had to be euthanized prematurely. One had acute arthritis and the other suffered from cardiovascular collapse with evident pulmonary oedema.

### Genetic characterization of the vancomycin-resistant bacteria

**Figure 4.**
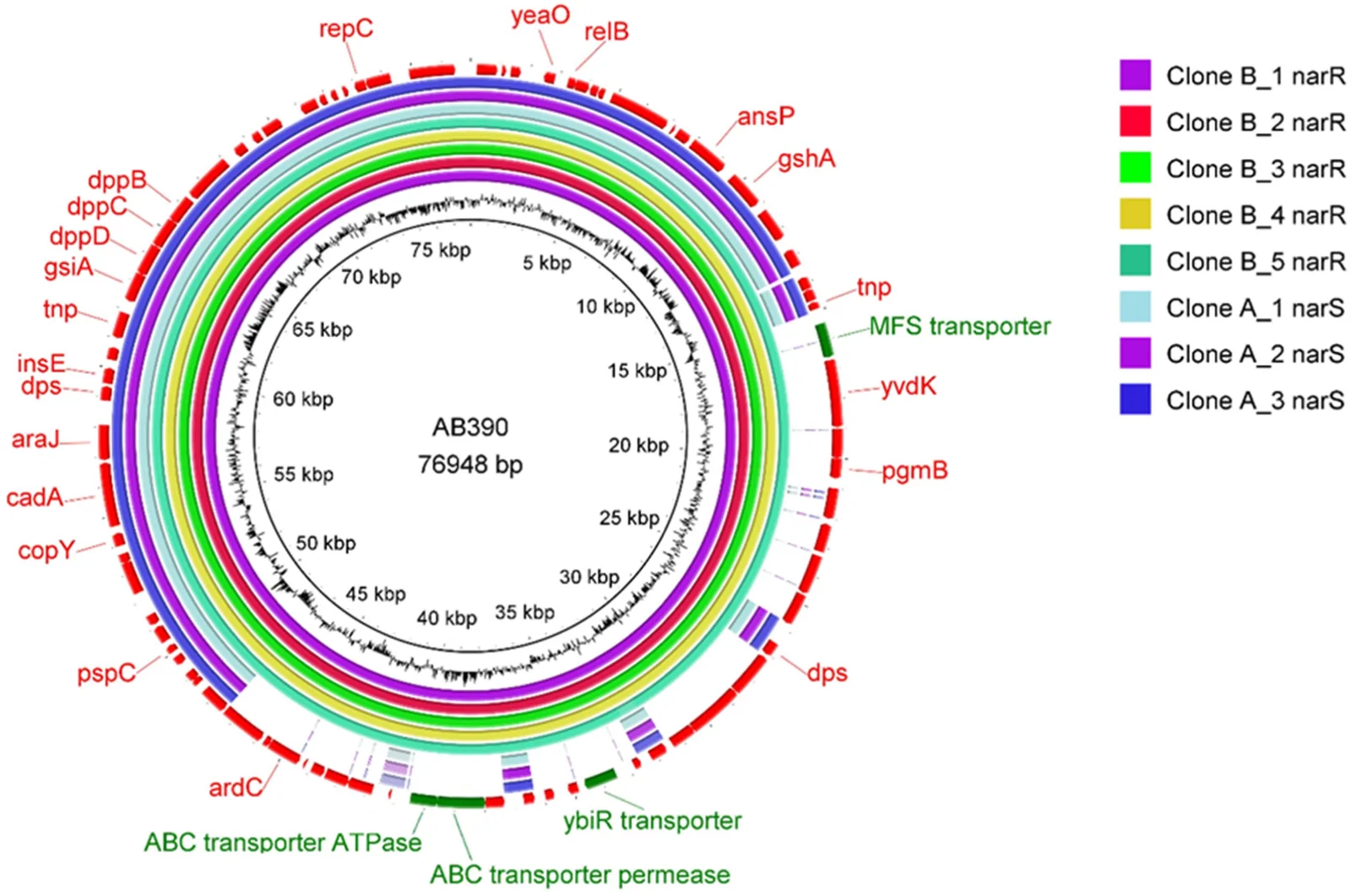
Comparative BLAST alignment of AB390 plasmids from *P. acidilactici isolates.* AB390 plasmids from narasin-susceptible *P. acidilactici* Clone A isolates, from broilers not treated with narasin in group 1, are compared with AB390 plasmids from *P. acidilactici* Clone B isolates with reduced susceptibility, from broilers fed narasin-supplemented feed in group 2. The plasmid sequence and annotation from Clone B_1 narR was used as reference. Annotations in green are transporters located within the region from 15 to 45 kbp, which is not present in Clone A isolates.

Colonies on SB agar with vancomycin or vancomycin and rifampicin appeared identical. To investigate the reason for the cfu discrepancies between the two types of selective agar, colonies were randomly selected from agar inoculated with caecal content. 12 colonies (five from group 1 and seven from group 2) were picked from SB agar supplemented with vancomycin and six colonies (two each from groups 3 and 6 and one each from groups 5 and 7) were picked from SB agar supplemented with vancomycin and rifampicin and the isolates were sequenced. The six isolates from groups 3, 5, 6 and 7 isolated from SB agar supplemented with vancomycin and rifampicin were all identical to the respective *E. faecium* inoculation strains (Table S3). Ten isolates from groups 1 and 2 were vancomycin-resistant *P. acidilactici* (Table S3). Group 1 isolates belonged to clone A whereas group 2 isolates belonged to clone B. Clone A and B differed by 20 000 SNPs. Two group 1 isolates were vancomycin-resistant *E. gallinarum* (Table S3). Broth microdilution showed that group 1 isolates were narasin susceptible, whereas five of seven group 2 isolates were narasin resistant (Table S3). Genome analysis did not detect the *narAB* genes in *E. gallinarum* or *P. acidilactici* (Table S3). However, a 36-gene region linked to narasin resistance on an AB390 plasmid, was present only in resistant *P. acidilactici* (Figure 4, Table S6). This region included three putative membrane transporters including ABC-type (distinct from NarAB), MFS-type and YbiR-family transporters.

## Discussion

The global rise in AMR infections poses a serious health threat.^58^ Stewardship across healthcare, dentistry, veterinary medicine and agriculture is vital to limit AMR.^59^ Understanding the consequences of antimicrobial use is key to effective control measures. We used *E. faecium* strains to study the role of NarAB-mediated ionophore resistance and in-feed ionophores in selecting VREfm in broilers. NarAB has been linked to resistance against tetracycline and erythromycin,^60^ suggesting that our results could be extrapolated to the possibility of narasin co-selecting for resistance to other clinically relevant antimicrobials, in addition to vancomycin, *in vivo*.

No rifampicin- and vancomycin-resistant bacteria were found in groups 1 and 2, confirming that resistance markers effectively distinguished inoculation strains from the resident microbiota and that cross-contamination did not occur. Detection of inoculation strains in groups 3 and 5 (feed without ionophores) suggests that vancomycin and narasin resistance do not severely impair colonization. This aligns with prior findings that although the pVEF plasmid imposes a fitness cost *in vitro*, VREfm can persist in the broiler gastrointestinal (GI) tract without antimicrobial selection pressure.^18,61^ It may also explain the presence, but gradual decline, of narasin-resistant *E. faecium* in Norwegian broilers after narasin use ended in 2015–2016.^19,23^

Presence of TC233 (narasin resistant) and absence of TC172 (narasin susceptible) in caecal content of broilers fed narasin confirms that the narasin resistance mechanism is required for selection of VREfm under narasin pressure. Declining cfu counts of TC172 in faeces indicates initial colonization followed by outcompetition by the resident microbiota. Similarly, the cfu count of WT1428-R was lower in narasin-fed broilers than in those on ionophore-free feed. These findings show that narasin resistance is crucial for stable *E. faecium* colonization in the presence of narasin.

Antimicrobial pressure favours resistant bacteria, altering the microbiota. Ionophores target Gram-positive bacteria, while Gram-negatives are naturally resistant.^62^ We proposed that ionophores would reduce Gram-positive bacteria in broilers, but no significant reduction was observed. Unexpectedly, vancomycin-resistant bacteria were found in all broiler groups, including those not inoculated. Genetic analysis revealed *P. acidilactici* and *E. gallinarum* carrying chromosomal vancomycin resistance. These strains were not detected in swabs from newly hatched broilers and feed and it is unclear where these bacteria originated from. However, vancomycin-resistant *P. acidilactici* and *E. gallinarum* have previously been isolated from broilers^63,64^ and may be part of the normal microbiota inherited from the parents via eggshells.

In ionophore-free groups, although undetectable in swabs of newly hatched broilers, both resident vancomycin-resistant bacteria and inoculation strains were equally present in caecal content of broilers. In narasin-fed groups, resident vancomycin-resistant strains outcompeted inoculation strains, indicating better adaptation to the broiler GI tract and narasin pressure. *P. acidilactici* has previously been shown to outcompete VRE for intestinal colonization in a mouse model.^65^

Lasalocid at 19% of recommended inclusion rate did not select for vancomycin-resistant bacteria, correlating with previous findings that NarAB does not confer lasalocid resistance *in vitro.*^22^ However, we cannot exclude that the lasalocid concentration in the feed did not reach levels required for selection *in vivo*. Most *P. acidilactici* isolates from group 2 were narasin resistant whereas none from group 1 grew on narasin agar. Although the dataset is limited, our results indicate that narasin selects for a resistant resident microbiota. Narasin resistance in *P. acidilactici* correlated with a 36-gene plasmid region, including three putative membrane transporters, although their function remains unknown and requires further characterization.

This study corroborates the time series of epidemiological data from Swedish and Norwegian commercial broiler production showing that narasin selects for narasin-resistant enterococci and co-selects for VREfm.^19,23^ It should be noted that even in the presence of narasin, the VRE populations in Norway and Sweden have been declining over time, after the ban on avoparcin.^19,66^ However, there is a bias in information regarding ionophore resistance since AMR surveillance programmes have focused on ionophore resistance in *E. faecium.* Most *E. faecium* isolated from broilers are ionophore resistant, but vancomycin susceptible.^19,23,60^ Narasin- and vancomycin-resistant *E. cecorum* have previously been isolated from broilers^67^ and in this study we demonstrate the presence of vancomycin- and narasin-resistant *P. acidilactici*. This implies that use of in-feed narasin selects for a narasin-resistant microbiota of unknown size and composition which may suppress the VRE population over time. An inoculation strain exhibiting narasin resistance and vancomycin susceptibility was not included in this study, limiting our ability to assess whether such a strain would outcompete the vancomycin- and narasin-resistant inoculation strain.

AMR bacteria in livestock can expand into the community and healthcare system,^68^ and persistent VRE in poultry may pose a public health risk. It is important to note that failure to isolate VRE from broiler populations does not confirm its absence from the microbiota, but rather that the numbers are below the detection limit of the method used. In this study, inoculation strains constituted <1% of growth on SB agar without antimicrobials, making VRE detection unlikely by non-selective methods. We therefore propose that VRE are more prevalent in animal populations than currently reported.

It is sometimes argued that VRE of animal origin rarely cause severe infections in humans,^28,69^ and that strains of *E. faecium* may be host specific.^28,70–72^ However, it is not necessary for VRE of animal origin to infect humans to pose a threat to human health. VREfm from broilers have been shown to colonize the GI tract of broiler farmers,^73,74^ vancomycin resistance can transfer between enterococcal isolates of animal and human origins,^18,22,29,75^ and inter-species transfer of vancomycin resistance can occur between VRE and *Staphylococcus aureus.*^76,77^ This implies that transient colonization of humans by VRE of animal origin has the potential to pose a threat to human health, but the likelihood is unknown. In addition, *narAB* genes have been detected in VREfm isolated from human infections^72,78^ and a nationwide study revealed that *narAB-*containing isolates occur frequently among VREfm in Taiwanese hospitals.^78^ These recent studies indicate that vancomycin resistance originating from animal populations has spread into the human population. Furthermore, *P. acidilactici,* is an intrinsically vancomycin-resistant opportunistic pathogen that can cause severe infections in immunocompromised individuals,^79–81^ but it is currently not known whether selection of *P. acidilactici* in broilers fed narasin-supplemented feed poses a threat to human health. Narasin resistance has not been considered relevant for human medicine and it is likely that narasin resistance in bacterial isolates from human infections is underreported.

Our results justify a call for caution for the use of ionophores as feed additives and more research is needed to fully understand the risks for human health associated with prophylactic use of ionophores in livestock production.

## Supplementary Material

dkag253_Supplementary_Data

## Supplementary data

Tables S1 to S6 are available as Supplementary data at *JAC* Online.
